# Supplementary material for: Ammonia marine engine design for enhanced efficiency and reduced greenhouse gas emissions
Source: Nat Commun. 2024 Mar 7;15:2110. doi: 10.1038/s41467-024-46452-z (PMC10920916; doi:10.1038/s41467-024-46452-z)
Supplement: Supplementary file 1 — Supplementary Information [file 41467_2024_46452_MOESM1_ESM.pdf]

Supplementary information for Ammonia marine engine design for enhanced efficiency and reduced greenhouse gas emissions

Xinyi Zhou[1, 3], Tie Li[1, 2, *], Run Chen[1, 2], Yijie Wei[4], Xinran Wang[2], Ning Wang[2], Shiyan Li[1, 2], Min Kuang[5], Wenming Yang[3, *]

1. State Key Laboratory of Ocean Engineering, Shanghai Jiao Tong University, PR China
2. Institute of Power Plants and Automation, Shanghai Jiao Tong University, PR China
3. Department of Mechanical Engineering, National University of Singapore, Singapore
4. National Engineering Research Center of Special Equipment and Power System for Ship and Marine Engineering, PR China
5. Faculty of Maritime and Transportation, Ningbo University, PR China

*Corresponding author: 800 Dongchuan Rd., Shanghai, PR China, 200240;

E-mail: litie@sjtu.edu.cn

*Corresponding author: 9 Engineering Drive 1, Singapore, 117575;

E-mail: mpeywm@nus.edu.sg

Contents

Supplementary Table 1 | Engine specifications and experimental operating condition under various engine loads of the base engine without IRGR system for validating the chemical kinetic mechanism and numerical models

Supplementary Table 2 | Operating conditions for comparison of the base engine without IRGR system and IRGR engine under various diesel energetic ratios

Supplementary Fig. 1 | Comparison of apparent heat release rate and in-cylinder pressure of the base engine between the experiments and simulations using the ammonia/n-heptane chemical kinetic mechanisms listed in Table 2. (a) 50% engine load, (b) 75% engine load, (c) 85% engine load, (d) 100% engine load. (80% ammonia energetic ratio, 1000 rpm, 120 MPa diesel injection pressure and 318 K intake temperature). Source data are provided as a Source Data file.

Supplementary Table 1 | Engine specifications and experimental operating condition under various engine loads of the base engine without IRGR system for validating the chemical kinetic mechanism and numerical models

Parameters	Value
Engine specification	4 cylinder without IRGR
Bore × stroke (mm)	95 × 102
Nozzle hole diameter (mm) × number	0.127 × 8
Compression ratio	~17.5
Engine load (%)	50, 75, 85, 100
Diesel injection pressure (MPa)	120
Ammonia energetic ratio (%)	80
Ammonia supply pressure (MPa)	0.6
Intake temperature (K)	318 ± 3
Engine speed (rpm)	1000

Supplementary Table 2 | Operating conditions for comparison of the base engine without IRGR system and IRGR engine under various diesel energetic ratios

Parameters	Base engine without IRGR<br>Cylinders #1-4	IRGR engine<br>Cylinders #2-4	IRGR engine<br>Cylinder #1
[a] Total input energy per cylinder (J)	1717	1717	~1988
Overall excess air ratio	1.5	1.1	0.7
Diesel injection timing (°CA aTDC)	from -20 to 0	from -20 to 0	-6 (constant)
Diesel energetic ratio (%)	3, 10, 20		
Engine speed (rpm)	1000		
Diesel injection pressure (MPa)	120		
Intake temperature (K)	318		

[a] For the IRGR engine, the unburned fuel energy taken away by the exhaust gas from Cylinder #1 used for recirculation is excluded when calculating the input energy for Cylinder #1, instead, it is taken into account for the hydrogen-rich cylinders (i.e. Cylinders #2-4).

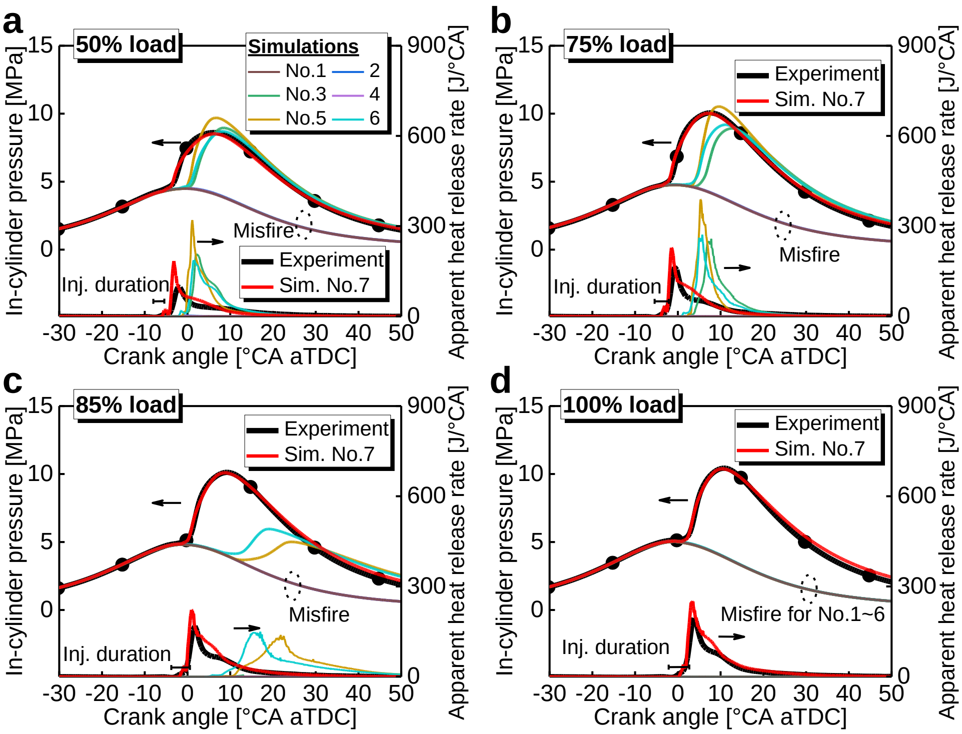

Supplementary Fig. 1 | Comparison of apparent heat release rate and in-cylinder pressure of the base engine between the experiments and simulations using the ammonia/n-heptane chemical kinetic mechanisms listed in Table 2. (a) 50% engine load, (b) 75% engine load, (c) 85% engine load, (d) 100% engine load. (80% ammonia energetic ratio, 1000 rpm, 120 MPa diesel injection pressure and 318 K intake temperature). Source data are provided as a Source Data file.
