# Supplementary material for: Ammonia marine engine design for enhanced efficiency and reduced greenhouse gas emissions
Source: Nat Commun. 2024 Mar 7;15:2110. doi: 10.1038/s41467-024-46452-z (PMC10920916; doi:10.1038/s41467-024-46452-z)
Supplement: Supplementary file 3 — Description of Additional Supplementary Files [file 41467_2024_46452_MOESM3_ESM.pdf]

Description of Additional Supplementary Files

File Name: Supplementary Code 1
Description: Mechanism file for the ammonia/n-heptane chemical kinetic mechanisms developed in this study.

File Name: Supplementary Code 2
Description: Thermal file for the ammonia/n-heptane chemical kinetic mechanisms developed in this study.
